# A Suburban Medical Lecture

**Published:** 1889-12-21

**Authors:** 


### The Doctor's Pulpit.

### A SUBURBAN MEDICAL LECTURE.

A few evenings ago a young medical man, full of the learning of the schools, discoursed for an hour in a suburban lecture-hall on the "Air We Breathe, and the Water We Drink." He had brought together a vast mass of common and uncommon scientific knowledge, which he poured out with unceasing flow from a legibly-written manuscript, to the evident amazement and even alarm of his unscientific audience. The common air he declared to be filled with living germs, germs which in unfavourable circumstances may increase and multiply at the expense of man and to the endangering of his life. He pointed out the situations and circumstances most conducive to the deterioration of the air, and the opposite conditions favourable to the increase of its purity and vital value. With less detail, though with not less alarming precision of statement, he expatiated on "impure water," and on the various dangers which attend the Londoner in his use of the beverage which Nature has provided for the quenching of his thirst. Once more the classical "Pump of Old Broad-street" was pressed into service; once more its limpid qualities were dilated upon, and once more the ghastly details of its death-dealing potency made the hair of a horrified audience stand on end.

To what purpose was all this scientific waste? Or was it truly and indeed waste? It is certain that the majority of the lecturer's audience did not understand the significance of one quarter of what was said, whilst even those who did understand were compelled to acknowledge to themselves how powerless they were in a vast city like London to make any useful application of scientific principles which, though so difficult to apply, are yet vital to life. The lecturer, the lecture, and the audience were quite typical of much of the scientific teaching that is being carried out in many of the large centres of population in this country at the present time. Is such scientific teaching of any practical value, or is it mere waste of time?

Undoubtedly, from the standpoint of the world's material and mental progress, any method of promulgating the facts and principles of science is to the general advantage. Many persons who hear such facts and principles for the first time may entirely fail to apprehend their real significance; some may even so distort them as to make them absolutely ridiculous and grotesque. But there will always be a few who will see the general drift of a teacher's meaning; and whose curiosity and interest being thus aroused, will not be content until they have mastered for themselves the full significance of all that has been taught. Moreover, the facts of science are well calculated to excite wonder and to arouse curiosity. The educational and disciplinary value of scientific acquirements is exceedingly great. Whilst science can never be a substitute for logic and ethics, for history and religion, in the training and development of the human mind, a general appreciation of scientific methods and results seems to be an absolute requisite for understanding and dealing rationally with the various practical problems, religious, social, and political, of our own times.

In many respects the progress of science may be compared with the growth and increasing influence of religion. Religion, with large numbers, is a mere fanaticism. It consists, to them, of one single apprehended principle or fact, which, though true in itself, is converted by disproportionate use into a dangerous superstition. Every distinct religious principle and doctrine requires to be kept in balance and harmony by the controlling influence of all the great principles in combination. It is not expected that every religious man and woman can apply this rule, or even understand that it exists. But it is certainly to be insisted upon that the teachers of religion shall have so much generalising power and capacity for original thought as is implied in the satisfying of such a demand as is here made. If the teachers of religion do not possess this luminous, controlling, and order-producing power of mind, they spread fanaticism and not reasonable religion. But though, as a result, the many become fanatics on the one hand or atheists on the other, there are always some who pierce through the crude masses of truth and falsehood presented to them, and extract for themselves wholesome and nourishing doctrine.

The case of science is exactly parallel. Not a few who are considered teachers of the sciences are mere human telephones. They hear the voice of authority, proclaiming certain facts and principles; and those facts and principles they do their best to impress upon their own memories, without having the power, by means of the mental digestive fluids of comparison, philosophy, and the like, to convert them into assimilable nourishment. Or, in less figurative language, they are conducting pipes, pure and simple, for other men's ideas. The result of this method of "teaching" is that science is considered difficult, unpractical, uninviting, and a nuisance. The pity is that many men who think themselves scientific, and who really do know something of science, encourage the public to consider scientific questions exceedingly stiff subjects to handle. Not long ago the writer had the opportunity of hearing a clergyman, the Rev. H. R. Haweis, lecture for an hour and a half on a profoundly scientific subject. His genius so lighted up the dark places and smoothed down the rough ones that the difficult became child's play, and the impossible delightful. That is the way to teach science. It is the way to teach everything. Teachers are always insisting that students and readers should work. Most true! But how much better a man or a boy will work if he is taught and helped to become master of every difficulty in his subject, instead of being compelled to exhaust his brains in vain attempts, and to pass by important principles uncomprehended and unsubdued.

Scientific lectures, suburban and otherwise, are too often mere compilations. A very little knowledge is sufficient to enable an industrious man to cull from books as much matter as will do for an hour's reading, and so to arrange it as to make it look like an original and extremely learned lecture. In too many cases it is not worth the paper it is written on. A small number of hearers, as we have said, may be able to extract intellectual and valuable food from it; the majority are merely confused and confounded, and left more hopelessly in the dark than before. If young medical men with leisure and aptitude for exposition would rid themselves of the boyish desire to appear extremely learned, and be content to be simply useful; if they would try to put themselves in the place of their hearers, and to explain and illustrate their teachings so as to make themselves understood by the least intellectual members of their audience, they would not only render a great public service, but would find their own minds stimulated and strengthened much more than they find them advantaged as the result of mere hard reading. No one learns so quickly or so thoroughly as the teacher, if he always observes the golden rule of teaching as he himself would desire to be taught.

